# Association between polymorphism of GSTP1, GSTT1, GSTM1 and CYP2E1 genes and susceptibility to benzene-induced hematotoxicity

**DOI:** 10.1007/s00204-017-2104-9

**Published:** 2017-12-04

**Authors:** Mohamad Amin Nourozi, Masoud Neghab, Javad Tavakkoly Bazzaz, Saharnaz Nejat, Yaser Mansoori, Seyed Jamaleddin Shahtaheri

**Affiliations:** 1 Department of Occupational Health Engineering, School of Public Health, Tehran University of Medical Sciences, Tehran, Iran; 2 Department of Occupational Health Engineering, Research Center for Health Sciences, Institute of Health, School of Health, Shiraz University of Medical Sciences, Shiraz, Iran; 3 Department of Medical Genetics, Faculty of Medicine, Tehran University of Medical Sciences, Tehran, Iran; 4 Department of Epidemiology and Biostatistics, School of Public Health, Knowledge Utilization Research Center (KURC), Tehran University of Medical Sciences, Tehran, Iran; 5 Non-communicable Diseases Research Center, Fasa University of Medical Sciences, Fasa, Iran

**Keywords:** Genetic polymorphism, Susceptibility to benzene-induced hematotoxicity, Petrochemical employees

## Abstract

Occupational exposure to benzene has been associated with leukemia, anemia, leukopenia, and thrombocytopenia. Genetic susceptibility to benzene toxicity in humans may be related to variations in benzene metabolizing genes. The main objective of this study was to ascertain whether polymorphism of GSTP1, GSTM1, GSTT1 and CYP2E1 genes might influence susceptibility to the adverse effects of benzene among employees of a petrochemical plant. In this cross-sectional study, 124 employees of a petrochemical plant who had been occupationally exposed to benzene and had one or more abnormal hematological parameter (cases) and 184 subjects with a similar exposure scenario, free from any abnormal hematological parameters (referent) were studied. Atmospheric concentrations of benzene were measured and GSTM1 and GSTT1 genotypes were evaluated using the multiplex polymerase chain reaction (PCR) technique. Additionally, GSTP1 and CYP2E1 genotypes were determined by PCR- restriction fragment length polymorphism (PCR–RFLP). The frequency of null GSTT1 genotype in cases was significantly higher than that of referent group **(**32.3 vs. 18.5%, OR 2.1, 95% CI 1.23–3.56, *p* = 0.004). The mean value of platelets in subjects with null GSTT1 genotype was significantly lower than that of individuals with positive GSTT1 genotype (*p* = 0.015). Conversely, the mean value of leukocytes was significantly higher in subjects with null GSTM1 genotype as compared to those with positive GSTM1 genotype (*p* = 0.026). Logistic regression analysis showed that, subjects with null GSTT1 genotype had a significantly higher risk for hematological disorders, as compared to those with positive GSTT1 genotype (OR 2.1, 95% CI 1.23–3.56). Moreover, subjects with both null GSTT1 and GSTM1 genotypes had a significantly higher risk for hematological disorders as compared to subjects with positive GSTT1 and GSTM1 genotypes (OR 2.35, 95% CI 1.14–4.8). The results of this study showed that, individuals carrying null GSTT1 or both null STT1 and GSTM1 genotypes had a higher risk and were more susceptible to benzene-induced hematological disorders.

## Introduction

Benzene has been classified as a human hematocarcinogen by International Agency for Research on Cancer (IARC) (IARC 2017). Acute exposure to high concentrations of benzene can affect the central nervous system and cause dizziness, headaches, and nausea (Edokpolo et al. 2015), while, chronic exposure to benzene causes leukemia, anemia, leukopenia, thrombocytopenia, acute myeloid leukemia (AML), acute lymphocytic leukemia (ALL), myelodysplastic syndrome, and non-hodgkin lymphoma (Kim et al. 2007; Wan et al. 2002; Schnattera et al. 2010; Swaen et al. 2010; Ye et al. 2015). Similarly, some studies have reported genotoxicity, increased levels of persistent chromosome aberrations and reproductive effects as a result of exposure to benzene (Edokpolo et al. 2015). Given the hematotoxic potential of benzene, complete blood counts (CBC) has been recognized as an easy and readily available screening tool for assessing the hematotoxicity of this compound (Tunsaringkarn et al. 2013). The occupational safety and health administration (OSHA), states that, CBCs should be monitored for workers exposed to benzene (OSHA 2012). Occupational exposures to benzene occur within the petrochemical plants, petroleum refineries (Edokpolo et al. 2015), petrol distribution, and in manufacturing industries that require aromatic solvents or glues that contain benzene such as rubber production, shoe manufacturing, and printing (Weisel 2010; Carrieri et al. 2012).

Once absorbed, benzene is oxidized by phase I enzymes, mainly cytochrome P4502E1(CYP2E1) in the liver to form benzene oxide. Spontaneous rearrangement of benzene oxide to phenol (PH) from non-enzymatic pathway, which can undergo another CYP oxidation (CYP2E1), results in the production of potentially toxic hydroquinone (HQ) and 1,4-benzoquinone. Benzene oxide can also be hydrolyzed by microsomal epoxide hydrolase (mEH) into benzene dihydrodiol that is then converted to catechol and toxic 1,2-benzoquinone. Detoxification of the quinones can occur via NAD(P)H:quinine oxidoreductase-1 (NQO1) or via Glutathione s-transferases (GSTs). Benzene oxide may also undergo ring opening from CYP to produce trans, trans-muconic acid (TTMA) or from GSTs to produce S-phenylmercapturic acid (SPMA). However, the conjugation of these benzene metabolites with glutathione operated by GSTs for the production of less toxic compounds or non-toxic derivatives are necessary to be excreted in urine (Dougherty et al. 2008; Jx et al. 2006; Rappaport et al. 2010).

The GSTs are phase II enzymes involved in the detoxification of xenobiotics and catalyze the conjugation reactions between glutathione and compounds containing an electrophilic center such as chemotherapeutic drugs, carcinogens, environmental pollutants, and other xenobiotics (He et al. 2014). The GST family consists of several gene subfamilies of which GSTP1, GSTM1, and GSTT1 are the most important ones (Singh et al. 2011). There are wide individual variations in the activity of these liver enzymes (Dougherty et al. 2008). Both GSTM1 and GSTT1 genes display deletion polymorphism. Homozygous deletions of GSTM1 gene (GSTM1-deficient genotype) and GSTT1 gene (GSTT1-deficient genotype) result in the absence of enzyme activity, while in GSTP1 genotype, a single nucleotide substitution (A to G), which causes the change of isoleucine to valine (Ile105val, rs1695), decreases the GSTP1 enzyme activity (Lin et al. 2008). A number of studies have indicated that, individuals with high levels of metabolic enzymes involved in oxidizing benzene to more toxic metabolites such as CYP2E1 and low levels of metabolic enzymes involved in detoxification pathways, such as GSTT1 and GSTM1 were less resistant to benzene toxicity (Wan et al. 2002). Both GSTT1 and GSTM1 are involved in the detoxification of benzene oxide to SPMA. According to the result of some studies, the SPMA levels of subjects with GSTT1-positive genotype were significantly higher compared to individuals with null GSTT1 genotype (Lin et al. 2008, Mansia et al. 2012).

Given the differences between frequencies of variant alleles among different geographical populations and wide individual variations in the activities of liver enzymes, in this cross-sectional study, the effects of genetic polymorphism of GSTs and CYP2E1 (rs3813867) genotypes on the risk of benzene-induced hematotoxicity among employees of a petrochemical plant were investigated.

## Materials and methods

### Study population

This cross-sectional study was conducted in a petrochemical company in the south of Iran. Subjects with ongoing occupational exposure to benzene for at least 2 consecutive years participated in the study. Individuals with glucose-6-phosphate dehydrogenase (G6PD) deficiency, hemophilia, minor thalassemia and history of exposure to other chemical with hematotoxic effects were excluded from the study. Similarly, individuals with chronic diseases such as diabetes, cardiovascular disease and hypertension were not included in this study. In the first phase of this study, CBC parameters including white blood cell count (WBC), WBC differential, red blood cell count (RBC), hemoglobin (HGB), hematocrit (HCT), mean corpuscular volume (MCV), Mean corpuscular hemoglobin (MCH), mean corpuscular hemoglobin concentration (MCHC), red cell distribution width (RDW) and platelet (PLT) count of all employees of a petrochemical plant occupationally exposed to benzene were measured. In the second phase of the study, subjects were divided into two groups. The first group included 124 subjects with one or more abnormal hematological parameters. These were selected as cases. The second group included 184 subjects with normal hematological parameters. These were selected as referent. Referent subjects were matched with cases as far as variables such as sex, age, level of exposure, length of exposure, job title, and smoking habits were concerned. Additionally, all subjects were interviewed and a questionnaire containing questions regarding demographic variables, medical history, smoking habits, alcohol consumption, drug consumption and occupation-related information (job category, job history, working hours/day, length of exposure (employment), occupational or non-occupational exposure to other chemicals) was completed for them. All participants signed an informed consent form before commencement of the study.

### Assessment of benzene exposure

For all subjects, sampling and analysis of benzene were conducted according to the National Institute for Occupational Safety and Health (NIOSH) method 1501, (NIOSH 2003), using activated charcoal sorbent tubes and a sampling pump, with a flow rate of 100–200 ml/min. Three personal air samples were collected at the breathing zone of each subject during different hours of shift. Similarly, from each administrative office, a sample from the ambient air at the breathing zone height was collected. The samples were transferred to the laboratory, where they were analyzed by NIOSH 1501 technique.

### Genotyping analyses

Peripheral blood samples were taken from all subjects. The Genomic DNA was extracted from the sample after sampling, using a commercially available kit according to the manufacturer’s instructions (Qiagen DNA extraction kit). Each DNA sample was stored at − 20 °C until analysis.

The GSTM1 and GSTT1 genetic polymorphisms were evaluated using the PCR technique as described by Singh et al. (2011) and Cheng et al. (2013). Primers for GSTM1 were F:5-GAACTCCCTGAAAAGCTAAAGC-3, and R:5-GTTGGGCTCAAATATACGGTGG-3, and those for GSTT1 were F:5-TTCCTTACTGGTCCTCACATCTC-3 and R:5-TCACCGGATCATGGCCAGCA-3. The β-globin locus was used as an internal control to avoid false negative readings. The absence of PCR product for GSTM1 or GSTT1 in the presence of the β-globin band was indicative of a null genotype for GSTM1 or GSTT1. Individuals with one or two copies of the relevant gene were classified as the “positive” genotype and those with homozygous deletions as the “null” genotype. Experiments were repeated at least twice using both of the standard genotyping protocols. Detection of the GSTP1 codon 105 Ile to Val polymorphism (Ile105val, rs1695) was performed by PCR–RFLP technique, as described previously (Cheng et al. 2013). DNA templates were amplified with the primers: 5-CTTCCACGCACATCCTCTTCC-3 (upstream) and 5-AAGCCCCTTTCTTT TTCAGC-3 (downstream). Briefly, for the CYP450 2E1 gene analysis (rs3813867), any RFLP was detected by differences in PstI sites in the 5-flanking region following PCR amplification, using methods described by Hayashi et al. (1991) and Hsieh et al. (2007). The primers used for the amplification of CYP2E1 gene were 5-CCA GTC GAG TCT ACA TTG TCA-3 and 5-TTC ATT CTG TCT TCT AAC TGG-3.

### Statistical analyses

The normality of the distributions of data was assessed by the Kolmogorov–Smironov test. Independent sample *t* test was used to examine the statistical significance of the differences in quantitative variables between the two groups, when Kolmogorov–Smironov test showed that dependent variable was normally distributed. In contrast, Mann–Whitney *U* test was used when the dependent variable was not normally distributed. Categorical data were presented as frequencies (percentage). Statistical differences between categorical variables, genotype/allele frequencies, were investigated by *χ* ^2^ test or Fisher’s exact test. Logistic regression tests were used to determine the independent effect of polymorphism genotypes on the risk of blood disorders. The association was expressed as an OR at 95% CI. *P* value < 0.05 was considered to be statistically significant.

## Results

The demographic characteristics of subjects and the mean concentrations of benzene are presented in Table 1. All subjects were male. No significant differences were noted between both groups as far as demographic variables, exposure history and smoking habits were concerned. The arithmetic means of benzene concentration in the breathing zone of cases and referent subjects were 0.1 and 0.12 ppm, respectively.

**Table 1:** Demographic characteristics of subjects and benzene concentrations

Variable	Cases (*n* = 124)	Referent(*n* = 184)	*P* value
Age (year), (mean ± SD)	34.26 ± 6.64	34.3 ± 5.723	0.593*
BM kg/m^2^ (mean ± SD)	25.42 ± 2.85	25.59 ± 4.8	0.386*
Length of exposure (year), (mean ± SD)	7.3 ± 4.05	8.04 ± 4. 2	0.121*
Smoking: no (%)			
Yes	8 (6.5 0)	12 (6.5)	0.588^┼^
No	116 (93.5)	172 (93.5)	
Benzene concentration (ppm), (mean ± SD)	0.10 ± 0.195	0.12 ± 0.284	0.631*

*Independent sample *t* test

^┼^Chi-square test

Table 2 depicts the frequency of GSTP1, GSTT1, GSTM1 and CYP2E1 genotypes in both groups. The frequency of null GSTT1 genotype in cases was significantly higher than that of referent group (32.3 vs. 18.5%, OR 2.1, 95% CI 1.23–3.56, *p* = 0.004). Additionally, the frequencies of null GSTM1 and GSTP G/G genotypes in cases was higher than that of referent group, although the difference did not reach statistical significance.

**Table 2:** Frequencies of genetic polymorphism of GSTP1, GSTM1, GSTT1 and CYP2E1 in the studied groups

Genotype	Cases (*n* = 124)	Referent (*n* = 184)	OR (95% CI)	*P* value
GSTP1				
AA	62 (50)	81 (44)	–	0.214
AG	52 (41.9)	94 (51.1)		
GG	10 (8.1)	9 (4.9)		
GSTT1				
Positive	84 (67.7)	150 (81.5)	2.1 (1.23–3.56)	0.004
Null	40 (32.3)	34 (18.5)		
GSTM1				
Positive	60 (48.4)	96 (52.2)	1.16 (0.73–1.83)	0.296
Null	64 (51.6)	88 (47.8)		
CYP2E1				
C1C1	124 (100)	178 (97.3)	0.589 (0.94–0.99)	0.074
C1C2	0 (0)	5 (2.7)		

Table 3 exhibits the association between GST genotypes and hematological parameters in the study population. As only few individuals had the GSTP1 G/G genotype, those with GSTP1 A/G and G/G genotypes were combined. The mean value of platelets in subjects with null GSTT1 was significantly lower than that of individuals with positive GSTT1 genotype (*p* = 0.015). Conversely, mean value of leukocytes was significantly higher in subjects with null GSTM1 as compared to those with positive GSTM1 (*p* = 0.026). However, there was no statistically significant association between GST genotypes and other hematological parameters.

**Table 3:** The association between GST genotypes and hematological parameters in the study population

Blood parameters	GSTP1			GSTT1			GSTM1		
	AA *n* = 143	AG + GG *n* = 165	MD%	Positive *n* = 234	Null *n* = 74	MD %	Positive *n* = 156	Null *n* = 152	MD%
WBC	7.013 ± 1.75	7.057 ± 1.75	0.62%	7.028 ± 1.69	7.062 ± 1.62	0.48%	6.808 ± 1.54^a^	7.270 ± 1.77	6.78%
RBC	4.98 ± 0.196	5.028 ± 0.48	0.96%	4.99 ± 0.452	5.06 ± 0.508	1.4%	4.97 ± 0.42	5.043 ± 0.503	1.46%
HB	15.00 ± 1.19	15.12 ± 1.24	0.8%	15.068 ± 1.18	15.081 ± 1.33	0.08%	15.055 ± 1.25	15.087 ± 1.17	0.21%
HCT	43.86 ± 3.28	43.80 ± 2.65	0.13%	43.732 ± 3.03	44.164 ± 2.67	0.98%	43.735 ± 3.23	43.938 ± 2.65	0.46%
MCV	87.61 ± 7.93	87.67 ± 6.5	0.068%	87.858 ± 6.14	86.964 ± 9.83	1.02%	87.710 ± 7.44	87.576 ± 6.94	0.15%
MCH	30.38 ± 2.76	30.26 ± 3.05	0.39%	30.419 ± 2.69	30.015 ± 3.52	1.34%	30.434 ± 2.53	30.207 ± 3.26	0.75%
MCHC	34.38 ± 1.29	34.82 ± 4.29	1.27%	34.765 ± 3.62	34.142 ± 1.58	1.82%	34.442 ± 1.28	34.793 ± 4.45	1.02%
RDW	12.20 ± 0.60	12.22 ± 0.76	0.16%	12.222 ± 0.72	12.200 ± 0.60	0.18%	12.230 ± 0.78	12.203 ± 0.59	0.22%
PLT	206.35 ± 39.7	209.96 ± 42.07	1.74%	211.85 ± 40.5	196.94 ± 40.452^a^	7.57%	205.83 ± 36.8	210.78 ± 44.7	2.4%
LY	41.41 ± 9.7	40.72 ± 8.35	1.69%	40.698 ± 8.75	42.156 ± 9.87	3.58%	41.278 ± 8.54	40.812 ± 9.5	1.14%
MO	2.9 ± 1.6	3.05 ± 1.7	5.17	2.978 ± 1.63	3.021 ± 1.85	1.44%	3.119 ± 1.75	2.855 ± 1.6	9.24%
GR	54.75 ± 10.1	55.32 ± 8.8	1.04%	55.328 ± 9.22	54.215 ± 10.08	2.05%	54.458 ± 9.15	55.677 ± 9.69	2.24%

*MD* mean differences between two groups %

^a^Significantly different (independent sample *t* test, *p* < 0.05)

Age, smoking, job category, length of exposure and level of exposure to benzene were associated with blood cell counts. Therefore, these parameters were entered into the linear regression model. Results showed that, after adjusting for these confounders, significant positive associations exist between GSTM1 and WBC counts and significant negative associations between GSTT1 and PLT counts (Table 4).

**Table 4:** Association between GSTT1 and GSTM1 genotypes and hematological parameters using the linear regression model

Dependent variable	GSTT1			GSTM1		
	Beta	SE	*P*	Beta	SE	*P*
WBC	− 0.023	0.227	0.92	0.526	0.194	0.007^*^
RBC	0.08	0.064	0.21	0.063	0.056	0.25
HB	0.006	0.167	0.97	0.012	0.144	0.93
HCT	0.487	0.406	0.23	0.119	0.352	0.73
MCV	− 0.99	0.99	0.31	− 0.116	0.864	0.89
MCH	− 0.047	0.40	0.24	− 0.256	0.348	0.46
MCHC	− 0.068	0.45	0.13	0.361	0.393	0.35
RDW	− 0.028	0.09	0.75	− 0.009	0.077	0.90
PLT	− 12.025	5.63	0.034^*^	5.51	4.89	0.26
LY	1.75	1.22	0.15	− 0.056	1.064	0.59
MO	0.054	0.23	0.81	− 0.212	0.203	0.29
GR	− 1.42	− 1.29	0.27	1.29	1.11	0.247

^*^ *p* < 0.05 statistically significant

Logistic regression model was used to analyze the association between GST genotypes and risk of benzene-induced hematological disorders. Table 5, illustrates the results of this analysis. As shown, subjects with null GSTT1 had a significantly higher risk for hematological disorders, as compared to those with positive GSTT1 polymorphism (OR 2.1, 95% CI 1.23–3.56). Similarly, subjects with both null GSTT1 and GSTM1 had a significantly higher risk for hematological disorders as compared to subjects with positive GSTT1 and GSTM1 genotypes (OR 2.35, 95% CI 1.14–4.8). No significant differences were noted in the risk of hematological disorders among GSTP1 and GSTM1 genotypes.

**Table 5:** Risk of hematological disorders in the studied groups

	*B*	SE	Crude OR (95% CI)	Adjusted OR (95% CI)
GSTP GG	− 0.325	0.242	0.723 (0.45–1.16)	0.736 (0.45–1.19)
GSTP AG	0.373	0.489	1.45 (0.55–3.78)	1.069 (0.38–2.98)
GSTP AA^a^			1 (Ref)^a^	
GSTT1 null	0.742	0.27	2.1 (1.23–3.56)^a^	2.27^a^ (1.31–3.93)
GSTT positive^a^			1 (Ref)^a^	
GSTM1 null	0.152	0.233	1.16 (0.73–1.8)	1.068 (0.66–1.7)
GSTM1positive^a^			1 (Ref)^a^	
GSTM1–GSTT1 (−,−)	0.855	0.366	2.35 (1.14–4.8)^a^	2.26 (1.08–4.7)^a^
GSTM1–GSTT1 (+, −)(−, +)	0.211	0.254	1.23 (0.75–2.03)	1.22 (0.72–2.04)
GSTM1, GSTT1^a^ (+, +)			1 (Ref)^a^	

*OR* Odds ratio, *95% CI* 95% confidence interval

^a^Referent group, *p* < 0.05 considered statistically significant; Logistic regression model, OR adjusted for age, smoking, job category, length of exposure and levels of exposure to benzene

## Discussion

Benzene is known to possess hematoxic potentials at high concentrations resulting in significant decreases in white blood cell, red blood cell, and platelet counts. However, the results of different studies, particularly, regarding exposure to low levels of benzene are not similar. Several studies have found significant associations between benzene exposure and hematological effects. Lan et al. reported decreased WBC, granulocytes, lymphocytes, B cells and platelets in Chinese workers occupationally exposed to benzene at air levels of 1 ppm or less (Lan et al. 2004). Similarly, Qu et al. reported a decrease in RBC, WBC and neutrophils at exposure to 0.25 ppm of benzene or less in Chinese workers (Qu et al. 2002). Likewise, Ye et al. reported decreased WBC in Chinese workers occupationally exposed to low levels of benzene (equal to 1 ppm) (Ye et al. 2015). Conversely, in some studies, no significant decreases have been observed in CBC parameters as a result of exposure to low levels of benzene. For instance, in the study of Swaen et al. performed on a group of workers of a chemical plant in Netherlands, no significant differences were noted between the hematological parameters of subjects with different exposure levels (below 0.5 ppm, 0.5–1 ppm, above 1 ppm), or exposed employees compared with unexposed group (Swaen et al. 2010). Similarly, in a study conducted by Tsai et al. on 1200 workers in the US petrochemical industries, with a mean exposure level of 0.6 ppm benzene from 1977 to 1988 and 0.14 ppm afterwards, no increased abnormalities were found in hematological parameters of exposed employees (Tsai et al. 2004). Similarly, in the study of Collins et al. on 387 benzene-exposed workers (mean concentration 0.55 ppm) and 553 unexposed workers, no significant differences were noted between lymphocyte counts of both groups (Collins et al. 1997). It has been speculated that genetic polymorphism of the enzymes responsible for benzene metabolism such as CYP and GSTs predisposes some individuals to benzene toxicity (Kim et al. 2007). However, there are differences in the frequencies of variant alleles between different geographic populations (Dougherty et al. 2008). In the current study, the frequencies of null GSTT1 and GSTM1 were 24 and 49.4 percent, respectively (data not shown), which is similar to those found in a previous study conducted on seven different Iranian populations (Nasseri et al. 2015). In China, GSTT1 allele frequencies including positive and null genotype were 40 and 60%, respectively. While, in Caucasian population including European and Caucasian Americans, GSTT1 null allele frequency was 17.5% (Lin et al. 2008). Conversely, the frequency of null GSTM1 genotype was 32.0–53.5% in Asians, 44.1–57.8% in Europeans and 27.6–56.9% in Americans (Ye and Song 2005). Our data showed that, the frequency of null GSTT1, GSTM1 and GSTP GG in cases was higher compared to the referent group, although no significant differences were found between two groups for GSTP1, GSTM1 and CYP2E1 genotypes. Among the GSTs, both GSTM1 and GSTT1 affect the production of SPMA, although the role of GSTT1 is more important (Kim et al. 2007). Urinary levels of SPMA in subjects with positive GSTT1 were significantly higher than those of subjects with null GSTT1 genotype (Lin et al. 2008; Mansia et al. 2012). These results support the hypothesis that in subjects with positive GSTT1 genotype, higher amounts of benzene are converted to SPMA and subjects with the null GSTT1 are less resistant to benzene toxicity.

In the present study, PLT counts in subjects with null GSTT1 was significantly lower compared to the positive GSTT1 genotype (*p* = 0.015). Conversely WBC counts was significantly higher in subjects with null GSTM1 as compared to positive GSTM1 (*p* = 0.026). However, there was no statistically significant association between GST genotypes and other hematological parameters. A similar pattern was noted after adjusting for confounders such as age, smoking, job category, job history, and exposure to benzene. In the study of Saadat also WBC count increased in subjects exposed to natural sour gas who had null genotypes of GSTM1 and GSTT1 (Saadat 2004). Similarly, Hsieh et al. found that individuals with both positive GSTT1 and GSTM1 genotypes showed the highest prevalence of low WBC when the benzene exposure was high (Hsieh et al. 1999). Conversely, Ye et al. reported lower WBC counts in workers with null GSTT1 and null GSTM1 genotypes (Ye et al. 2015). However, apart from GSTs, numerous other enzymes are also involved in the metabolism of benzene. For instance, apart from CYP2E1 and GSTs, epoxide hydrolase (EPHX), NQO1 and MPO are involved in metabolism of benzene (Dougherty et al. 2008). Some studies have indicated that, benzene metabolites individually or collectively cause benzene toxicity (Ye et al. 2015). This apparent discrepancy could be explained by the fact that apart from GSTs, numerous other enzymes such as epoxide hydrolase (EPHX), NQO1 and MPO are also involved in the metabolism of benzene (Dougherty et al. 2008). Consequently, toxic effects of benzene are thought to be the results of overall interaction of all enzymes involved in the benzene biotransformation pathways. Therefore, the difference in the results of these studies may be due to differences in genetic polymorphism of these enzymes between different populations and wide individual variations in the activity of liver enzymes.

Logistic regression analysis revealed that subjects with null GSTT1 genotype had a significantly higher risk of hematological disorders as compared to those with positive GSTT1 polymorphism (OR = 2.1, CI = 1.23–3.56). Moreover, the result showed that, subjects carrying both null GSTT1 and GSTM1 genotypes had higher risk of hematological disorders compared to subjects with both positive GSTT1 and GSTM1 genotypes (OR = 2.35, CI = 1.14–4.8). However, there was little variation in OR values, even after adjustment for confounders such as age, smoking, job category, length of exposure and levels of exposure to benzene. Several previous studies focused on a possible association between the genetic polymorphism of GSTM1 and GSTT1 genes and the risk of blood disorders in persons occupationally exposed to benzene. In accordance with our results, Wan et al. reported 4.5-fold increase in the risk of benzene poisoning among workers with null GSTT1 genotype (95% CI = 1.13–17.54) compared to the workers with positive GSTT1 genotype (JX et al. 2006). Similarly, Bhat et al. reported higher frequency of chronic myeloid leukemia in individuals with null GSTT1 compared to GSTT1-positive carriers (OR: 2.12; 95% CI: 1.12–4.02), (Bhat et al. 2012). In a meta-analysis, it was revealed that, the GSTM1-null genotype, but not GSTP1 Ile105Val polymorphism, was associated with an increased risk of acute myeloid leukemia in East Asians and GSTT1-null genotype in Caucasians. In addition, the presence of the double-null genotypes increased the risk of acute myeloid leukemia in both Caucasians and East Asians (He et al. 2014).

In this study, no significant effects or associations were found between CYP2E1 alleles and hematological disorders. Most studies also have failed to find any effect of CYP2E1 on biomarkers of exposure or effect. This may be due to highly polymorphic nature of this gene which leads to a wide range of enzyme activity levels between individuals and considerable inter-individual variability in human CYP2E1 activity (Dougherty et al. 2008; Marchand et al. 1999).

The results of this study showed that, the exposed workers carrying null GSTT1 or both null GSTT1 and GSTM1 genotype had higher risk of hematological disorders and support the hypothesis that subjects with GSTT1 and GSTM1 null genotype would be less resistant to benzene toxicity and are considered as subjects with increased risk of blood disorders.

The main limitation of our study was that, genetic polymorphism of all enzymes in biotransformation pathway of benzene such as MPO, EPHX and NQO1 were not investigated. Therefore, further studies with larger sample sizes, and employees with more clinical and para-clinical findings from occupational exposure to benzene are required to overcome these limitations. Additionally, it is suggested that, all genetic polymorphisms of enzymes, involved in benzene metabolism to be investigated to further confirm these initial observations.
